# Poem by Asha

**DOI:** 10.4103/0019-5545.42406

**Published:** 2008

**Authors:** M. R. Asha

**Affiliations:** # 788/160, 18th cross, Ramanuja Road, Mysore - 570004, Karnataka, India. E-mail: ambleewine@yahoo.co.in

**Dedicated to special children…**

## TRYING TO BE…

Understand the challenges I face
Stop pushing me into the rat race
Give me the gift of time and space
And freedom to learn at my pace.

Frustrated anger and tears of guilt
Haunt me day in and day out
Upon which my self-esteem is built
In a desperate search for a way out.

Toys are meant to be broken
Don't hoard them in showcases of ignorance
Joys are meant to be spoken
Don't hide them in prisons of silence

Hurt and humiliated, seeking care,
When I reach out,
Just be there, to share,
Don't cancel me out.

Children are not parents' images
Nor, paradable trophies.
Instead of chasing mirages,
Accept and love WHAT IS.

Your smile and warm hug mean a lot to me
In the endless struggle of “TRYING TO BE”
Although imperfect, I'M A BEAUTIFUL SOUL
LOVING, UNIQUE, ETERNAL, AND WHOLE!

Dr. M. R. Asha is a freelance writer, Reiki Practitioner and researcher. Areas of her interest include Energy medicine, Neurolinguistic programming (NLP), Epigenetics, Psychoneuroimmunology, Parapsychology, Sensory neurobiology and Consciousness Research.

[ED.: *This poem was written in response to the editorial* ‘Wake up call from stars on the ground’ Indian J Psychiatry 50(1) Page No. 2 - 4.]

